# Hot liquid extrusion assisted drug-cyclodextrin complexation: a novel continuous manufacturing method for solubility and bioavailability enhancement of drugs

**DOI:** 10.1007/s13346-020-00854-w

**Published:** 2020-09-21

**Authors:** Alekhya Sri Nagini Manne, Aswathi R Hegde, Sushil Yadaorao Raut, Rajat Radhakrishna Rao, Vijay Induvadan Kulkarni, Srinivas Mutalik

**Affiliations:** 1 Department of Pharmaceutics, Manipal College of Pharmaceutical Sciences, Manipal Academy of Higher Education, Manipal, 576104 Karnataka India; 2 STEER Life India Private Limited, No. 290, 4th Main, 4th Phase, Peenya Industrial Area, Bangalore, 560058 Karnataka India

**Keywords:** Hot liquid extrusion, Carbamazepine, Cyclodextrin, Dissolution, Pharmacokinetics

## Abstract

In this study, drug-cyclodextrin (CD) complexes were prepared using hot liquid extrusion (HLE) process with an aim to improve solubility and bioavailability of carbamazepine. Saturation solubility studies of CBZ in water and different pH media showed a pH-independent solubility. Phase solubility studies of CBZ at different molar concentrations of beta-cyclodextrin (β-CD) and hydroxypropyl beta-cyclodextrin (HP-β-CD) indicated A_L_-type solubility profile with stability constants of 574 M^−1^ and 899 M^−1^ for β-CD and HP-β-CD. Drug-β-CD and drug-HP-β-CD complexes were prepared using HLE process and conventional methods (such as physical mixture, kneading method, and solvent evaporation) as well. Optimized complexes prepared using HLE *viz*. CBP-4 and CHP-2 showed a solubility of 4.27 ± 0.09 mg/mL and 6.39 ± 0.09 mg/mL as compared to plain CBZ (0.140 ± 0.007 mg/mL). Formation of drug-CD inclusion complexes was confirmed using DSC, FTIR, and XRD studies. Drug release studies indicated highest release of CBZ from CHP-2 (98.69 ± 2.96%) compared to CBP-4 (82.64 ± 2.45%) and plain drug (13.47 ± 0.54%). Complexes prepared using kneading showed significantly lesser drug release (KMB 75.52 ± 2.68% and KMH 85.59 ± 2.80%) as that of CHP-2 and CBP-4. Pre-clinical pharmacokinetic studies in Wistar rats indicated a significant increase in *C*_max_, *T*_max_, AUC, and mean residence time for CHP-2 compared to KMH and plain CBZ. All these results suggest that HLE is an effective method to increase the solubility of poorly water-soluble drugs.

**Electronic supplementary material:**

The online version of this article (10.1007/s13346-020-00854-w) contains supplementary material, which is available to authorized users.

**Figure:**
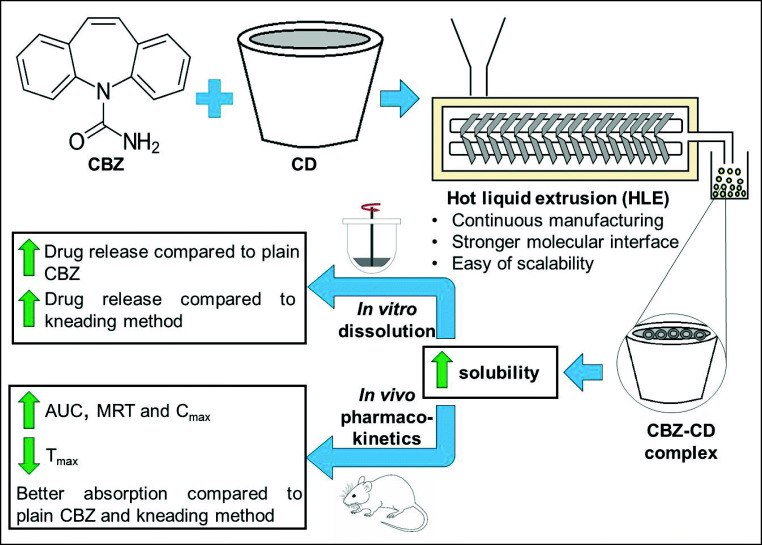
Graphical Abstract

## Introduction

Excessive discharge of electrical impulses in a group of brain cells leads to a neurological dysfunction, commonly called as seizures. Seizure episodes can range from a momentary lapse of attention to more severe prolonged convulsions. Recurrent unprovoked seizures or epilepsy is indicative of an underlying brain dysfunction. Epilepsy is a chronic disorder which may affect a part or the entire body and is generally accompanied by loss of cognition [1]. It is a common neurological condition that is prevalent in ~ 50 million people worldwide. Furthermore, about 80% of the people with epilepsy are found in developing countries [2]. The incidence of epilepsy is more prevalent during childhood, especially during the first few years of life [3] though it is not uncommon for people to develop the disease after the age of 60. Several causes such as defective brain development, abnormalities in brain structure, trauma, severe illness, stroke, infectious diseases, and genetics are predisposed to the development of epilepsy [4].

In the absence of a permanent cure for epilepsy, treatment strategies using anti-epileptic drugs are aimed at effectively controlling or managing the seizure episodes. Anti-epileptic drugs act by reducing the frequency and severity of seizures thereby diminishing the excessive electrical activity of the brain. Drugs act either by blocking the repetitive activation of sodium or calcium channels, augmenting the function of potassium channel, inhibiting excitation facilitated by glutamate, or stimulating GABA mediated inhibition [5].

Carbamazepine (CBZ), a dibenzazepine derivative, has widely been used in the treatment of epilepsy and neuropathic pain for the past five decades [6]. It acts by selectively binding to voltage-gated sodium channels and blocking its repetitive activation. However, oral administration of carbamazepine is marred by poor solubility of carbamazepine (~ 120 μg/mL) leading to lower bioavailability [7]. The variable absorption and low bioavailability can be ascribed to the slower dissolution rate of CBZ. Therefore, it is most appropriate to design a suitable approach to enhance the solubility of CBZ and hence improve the bioavailability.

Cyclodextrins (CDs), cyclic oligosaccharides connected to each other by α-(1, 4) bonds, have been routinely used to complex lipophilic drugs to overcome low solubility of drugs by forming inclusion complexes [8]. Beta-cyclodextrins (β-CDs) are widely used due to their reasonable cavity size which can accommodate a wide variety of drugs and their ready availability [9]. However, its low solubility (~ 2 g/100 mL) in aqueous media restricts its application as a drug carrier. Therefore, chemically modified β-CD derivatives have been synthesized to offer high-water solubility (> 50 g/100 mL), increased inclusion ability, and minimal toxicity. A few examples of β-CD derivatives are methyl-β-CD, 2,6-di-o-methyl-β-CD, 2-hydroxypropyl-β-CD (HP-β-CD), sulfo butyl ether β-CDs, and maltosyl and glucosyl β-CDs. [10, 11]. Among these, HP-β-CD has good water solubility and amorphous nature and can be widely used in drug formulations [12].

Interaction of drug molecule and CDs leads to the formation of host-guest complexes where the lipophilic cavity of CDs (host molecule) creates an advantage for the entrapment of poorly aqueous soluble drugs (guest molecule). When CDs are added to aqueous solution, the polar water molecules get into the cavity of CDs. However, these are immediately replaced by the relatively less polar drug molecules [13]. Hydrogen bonding between drug and CDs, van der walls interaction, charge transfer reactions, and replacement of polar water molecules by the less polar guest molecules act as the driving forces for the formation of inclusion complexes [14].

Previously, different carrier systems containing CBZ have been formulated in a bid to improve its dissolution and bioavailability, among which complexation with HP-β-CD showed the highest extent of absorption [15]. Conventionally, various methods such as physical blending [16], co-precipitation [17], spray-drying [18], freeze-drying [19], kneading [20], and solvent evaporation [15, 21] have been used to form drug-CD complexes. The potential of CBZ-CD complexes in increasing the solubility and dissolution rate of CBZ has been well documented. Oral bioavailability of CBZ was significantly enhanced from its HP-β-CD inclusion complex as compared to pure CBZ [22]. Complexing CBZ with sulfobutyl ether_7_ β-cyclodextrin (SBE_7_ β-CD), a β-CD derivative having high aqueous solubility, by spray-drying technique demonstrated an increase in the anti-epileptic activity compared to orally administered CBZ suspension [23, 24]. Enhanced solubility of CBZ from CBZ-β-CD complexes was reported by Koester et al. [25] where faster dissolution was observed with spray-dried or freeze-dried CBZ-β-CD complexes as compared to their simple physical mixtures. Addition of hydrophilic polymers like HPMC to CBZ-CD complexes further enhanced the solubility of CBZ [26, 27]. But then again, all these reports are limited to preparation of CBZ-CD complexes by conventional methods, which require longer processing times, involve the addition of solvents, and are difficult to scale up. In addition, they are batch processes and often lead to low yield [28].

Over the years, hot melt extrusion has emerged as an effective method to prepare pharmaceutical products like granules, pellets, sustained release tablets, and transdermal-related products and implants [29]. A hot melt extruder directs the raw materials into an instrument consisting of one or two rotating screws which are subjected to different temperatures, after which the processed material passes through a die to produce a material of required form and size.

Twin-screw-type extruders produce consistent and uniform mixing of different ingredients is essential in pharmaceutical preparations. Twin-screw extruders possess two screws which are placed side-by-side and the intermeshing provided by the rotating screws produces a homogenous material containing finely dispersed particles [30]. This further improves the rate of dissolution and hence enhances the bioavailability of drugs having poor aqueous solubility. In addition, inter- and intra-batch variability can also be minimized [29]. The screw rotation in the twin-screw extruder may be co-rotating (same) or counter rotating (opposite). Co-rotating twin-screw extruders offer advantages of proper mixing since high screw speeds and increased outputs can be achieved while maintaining adequate mixing and appropriate conveying characteristics [31].

Few reports are available on the application of melt extrusion to improve the solubility and dissolution characteristics of CBZ by formulating as solid dispersions in polymers such as hydroxypropyl methyl cellulose [32], Soluplus®, polyvinyl pyrrolidone, and polyvinyl acetate [33]. Despite being a promising approach, the possibility of regaining the crystalline nature (from an amorphous state) is a serious concern. In addition, the nature of the carrier, i.e., the size of the polymeric chain and drug:polymer ratio, significantly affects the drug release [34].

Until now, there are no reports on the preparation of CBZ-CD inclusion complexes by hot liquid extrusion (HLE) using a melt extruder, which offers advantages like continuous manufacturing, reduced residence time, self-cleaning screw feature, flexibility, enhanced mixing efficiency, and less heat generation to name a few. In addition, hot extrusion allows for stronger intermolecular interface and yields itself to scalability and commercial manufacture [35]. In the present study, we hypothesized that the solubility and hence the bioavailability of carbamazepine (CBZ) could be improved by forming drug-CD complexes using hot liquid extrusion technique (HLE). HLE is a continuous process and allows intimate mixing and stronger intermolecular interface compared to conventional complexation methods like kneading. The rationale behind this study was to assess the usefulness of HLE over conventional method in enhancing the solubility and bioavailability of CBZ. Hence, we prepared CBZ-CD complexes by HLE using a twin-screw melt extruder, and evaluated the effect of process parameters on the solubility and dissolution rate of drug-CD complexes. CBZ-CD complexes were also prepared by conventional methods and the solubility profiles compared against the complexes prepared by HLE process. All the complexes were evaluated with respect to saturation solubility, dissolution rate, DSC, FTIR, etc. Optimized complexes were subjected to pre-clinical in vivo pharmacokinetics study in order to assess the absorption rate of the CBZ.

## Materials and methods

### Materials

Carbamazepine was a generous gift from Sun Pharmaceutical Industries Ltd., Vadodara, India. Beta-cyclodextrin (β-CD) and hydroxypropyl-beta-cyclodextrin (HP-β-CD) were supplied by Steer Life India Pvt. Ltd., Bangalore, India, and Roquette Pharma, Mumbai, India. Acetonitrile (HPLC grade) was purchased from Finar Chemicals, Gujarat, India. Sodium dihydrogen phosphate was obtained from Merck Specialties Private Limited, Bengaluru, India. All other chemicals used in this study were of analytical grade.

### Animals

All animals were bred at the Central Animal Research Facility, Manipal Academy of Higher Education, Manipal. The experimental protocol was approved by the Institutional Animal Ethics Committee (IAEC), Kasturba Medical College, Manipal (Ref. No. IAEC/KMC/104/2017), and animal handling was carried out in accordance with the institutional and national guidelines for the care and use of animals. Adult female Wistar rats, weighing 200–250 g, were used for the in vivo experiments. They were kept at a temperature of 24–26 °C and adapted to a daily 12:12-h light:dark cycle (lights on at 6 A.M.). All the animals were given with standard rat feed and water ad libitum. One week prior to the experiments, all animals were handled daily to minimize the stress involved in the experimental procedure.

### Estimation of CBZ using HPLC

The amount of CBZ was estimated as per the method reported by Džodić et al. [36] using reverse-phase HPLC. The HPLC system (Shimadzu, Kyoto, Japan) comprised dual piston pumps, an auto sampler, a UV-visible detector, and LC solution software. Analysis was carried out on a reverse-phase C18 column (Kinetex®, 5 μm; 250 × 4.5 mm) maintained at 35 °C. Phosphate buffer solution (pH 7.0, 10 mM) was prepared by adding 1.5 g of sodium dihydrogen phosphate to 1000 mL of distilled water and the pH adjusted to 7.0 using 1 M sodium hydroxide solution. The buffer solution was filtered using a 0.22-μ membrane filter (Pall Pvt. Ltd., Bangalore, India). Mobile phase system consisted of a mixture of acetonitrile (ACN) and 10 mM phosphate buffer solution (30:70% v/v) and the flow rate was maintained at 1.5 mL/min. Before using, degassing of mobile phase was done using an ultrasonic bath sonicator (Medica Instrument Mfg. Co., Mumbai, India). Injection volume of the sample was 40 μL and analysis was performed at a wavelength of 285 nm. The total run time of the sample was 12 min.

In a similar manner, the estimation of CBZ in rat plasma was carried out [37] for pharmacokinetics study. Protein precipitation method was followed to extract CBZ from plasma. Briefly, an aliquot of 200 μL of plasma and 50 μL of propyl paraben (internal standard; 100 μg/mL in methanol) was mixed and vortexed for 2 min. The precipitating agent (chilled acetonitrile; 900 μL) was added after which the mixture was vortexed for 2 min. A cooling centrifuge (Sigma Laborzentrifugen GmbH, Germany) was used to centrifuge the samples for 10 min at 5000 rpm and 4 °C, following which the clear supernatant was injected into HPLC. Samples were eluted using a mobile phase mixture of acetonitrile and phosphate buffer (pH 7.0; 10 mM) (30:70% v/v) at a flow rate of 1.5 mL/min. The total run time of the sample was 15 min. The peak of CBZ was good in shape and well resolved from other peaks of plasma.

### Saturation solubility of CBZ

The solubility of carbamazepine in water or buffer solutions of varying pH *viz*. hydrochloric acid pH 1.2; acetate buffer pH 4.6; phosphate buffer pH 5.8, 6.8, and 7.4; and alkaline borate buffer pH 9.2 was determined according to the reported method [38]. Briefly, a surplus of CBZ was added in an Eppendorf tube containing water or different standard buffer solutions. Excess amount of drug was added to ensure that the drug was reaching saturation level. The tubes were then placed on Rotospin (TBS India, Telimatic and Biomedical Services Pvt. Ltd., Bangalore, India) and rotated at 50 rpm for 24 h at room temperature. The solutions were passed through 0.22-μ syringe filter and amount of CBZ dissolved was analyzed spectrophotometrically at 285 nm.

### Phase solubility studies

The method reported by Higuchi was used to carry out the phase solubility studies [39]. Varying concentrations (0–20 mM) of β-CD and HP-β-CD in water were prepared. A surplus of CBZ was added to β-CD or HP-β-CD solutions which were then stirred for 24 h in a Rotospin at room temperature. After achieving equilibrium, all samples were filtered and the absorbance was recorded at 285 nm using HPLC. Phase solubility diagrams were plotted by taking molar concentration of solubilized drug (CBZ) vs. the molar concentration of β-CD or HP-β-CD. The apparent stability constants (*K*_s_) were estimated from the straight line of the phase solubility diagrams, and the complexation efficiency was determined using the intercept method.

### Preparation and optimization of drug-cyclodextrin complexes

The drug-cyclodextrin complexes (CBZ-β-CD and CBZ-HP-β-CD) were prepared using various methods such as co-rotating twin-screw processing, physical mixture, solvent evaporation, and kneading. The complexes were evaluated with respect to solubility and compared with that of plain carbamazepine.

#### Co-rotating twin-screw extrusion method

Different batches of drug-cyclodextrin complexes (CBZ-β-CD and CBZ-HP-β-CD) were prepared using the co-rotating twin-screw hot melt extruder (Omicron 10P, STEER Engineering, Bangalore, India) having a screw diameter ratio of 1.71 and a length diameter ratio of 24. The extruder was fitted with a barrel having a diameter of 11 mm. Briefly, the co-rotating twin-screw processor consists of two screws rotating in the same direction in a temperature-controlled barrel which is heated. The feed is added to the hopper from where it moves to the barrel. At the end of the barrel, a die is attached to remove the product.

**Fig. 1:**
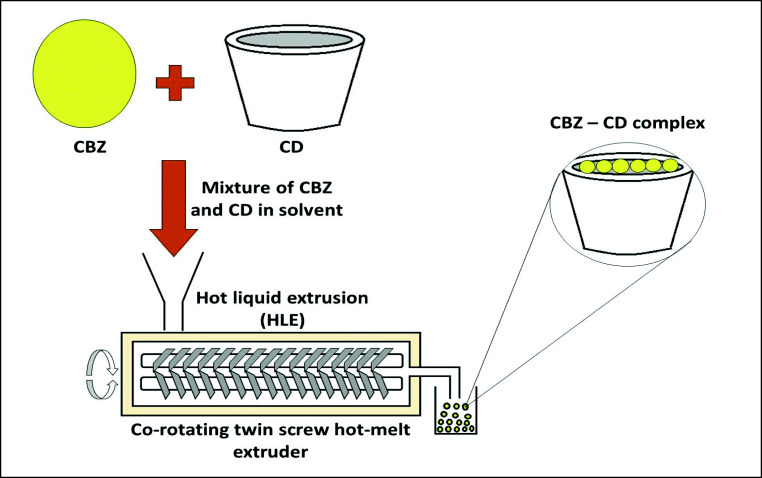
Schematic diagram from preparation of CBZ-CD complexes using HLE. CBZ—carbamazepine; CD—cyclodextrin; HLE—hot liquid extrusion

Initially, a mixture of drug and β-CD or HP-β-CD was prepared in 3–4 mL of solvent and introduced into the hopper of the processor using a gravimetric screw. The material was collected at the other end after being processed in the processor. The resulting aqueous dispersion was dried for 24 h at 50 °C. The schematic representation of the process is shown in Fig. 1. The drug-cyclodextrin complexes were optimized with respect to four process parameters *viz*. screw speed, solvent composition, temperature of barrel (B2, zone 2; B3, zone 3; and B4, zone 4), and molar ratio of drug and cyclodextrin. The effect of screw speed (25–250 rpm) of the processor was analyzed by keeping all other parameters constant. Different pH media such as distilled water, hydrochloric acid (pH 1.2), alkaline phosphate buffer (pH 4.0), acetate buffer (pH 4.6), neutralized phthalate buffer (pH 5.0), phosphate buffers (pH 5.8, 6.8, and 7.4), and alkaline borate buffer (pH 9.2) were used to determine the effect of solvent on the solubility of the prepared complexes. Effect of temperature on the solubility of prepared complexes was observed by varying the temperatures at different zones, *viz*., B2, B3, and B4 of the barrel (30–100 °C; B1 was set at a constant temperature of 32.5 °C). Different molar ratios of drug:cyclodextrin (1:0.5, 1:1, and 1:2) were used to determine the effect on solubility of complexes. Tables 1 and 2 show the composition and optimization results for CBZ-β-CD and CBZ-HP-β-CD complexes. The prepared complexes were then subjected to saturation solubility studies and compared against the saturation solubility of pure carbamazepine.

**Table 1:** Optimization of CBZ-β-CD complexes using HLE process

Batch	Drug:β-CD molar ratio	Screw speed (rpm)	Solvent composition	Temperature of barrel (°C)			Solubility (mg/mL)
				B2	B3	B4	
Optimization of screw speed							
CBS-1	1:1	25	Water	32	30	30	1.72 ± 0.04
CBS-2	1:1	35	Water	32	30	30	1.47 ± 0.01
CBS-3	1:1	50	Water	32	30	30	1.67 ± 0.03
CBS-4	1:1	65	Water	32	30	30	1.74 ± 0.04
CBS-5	1:1	80	Water	32	30	30	1.87 ± 0.03
CBS-6	1:1	100	Water	32	30	30	1.78 ± 0.01
CBS-7	1:1	150	Water	32	30	30	1.71 ± 0.01
CBS-8	1:1	200	Water	32	30	30	1.65 ± 0.01
CBS-9	1:1	250	Water	32	30	30	2.11 ± 0.01
Optimization of solvent composition							
CBS-5	1:1	80	Water	32	30	30	1.87 ± 0.03
CBP-1	1:1	80	Hydrochloric acid buffer pH 1.2	32	30	30	2.00 ± 0.01
CBP-2	1:1	80	Alkaline phosphate buffer pH 4.0	32	30	30	1.73 ± 0.008
CBP-3	1:1	80	Acetate buffer pH 4.6	32	30	30	2.18 ± 0.05
CBP-4	1:1	80	Neutralized phthalate buffer pH 5.0	32	30	30	4.27 ± 0.09
CBP-5	1:1	80	Phosphate buffer pH 5.8	32	30	30	1.84 ± 0.01
CBP-6	1:1	80	Phosphate buffer pH 6.8	32	30	30	1.87 ± 0.004
CBP-7	1:1	80	Phosphate buffer pH 7.4	32	30	30	1.75 ± 0.007
CBP-8	1:1	80	Alkaline borate buffer pH 9.2	32	30	30	1.84 ± 0.03
Optimization of temperature							
CBP-4	1:1	80	Neutralized phthalate buffer pH 5.0	32	30	30	4.27 ± 0.09
CBT-1	1:1	80	Neutralized phthalate buffer pH 5.0	40	40	40	2.24 ± 0.01
CBT-2	1:1	80	Neutralized phthalate buffer pH 5.0	50	50	50	2.06 ± 0.01
CBT-3	1:1	80	Neutralized phthalate buffer pH 5.0	60	60	60	1.94 ± 0.01
CBT-4	1:1	80	Neutralized phthalate buffer pH 5.0	70	70	70	2.15 ± 0.01
CBT-5	1:1	80	Neutralized phthalate buffer pH 5.0	80	80	80	2.01 ± 0.01
CBT-6	1:1	80	Neutralized phthalate buffer pH 5.0	90	90	90	2.31 ± 0.016
CBT-7	1:1	80	Neutralized phthalate buffer pH 5.0	100	100	100	2.15 ± 0.007
Optimization of molar ratio							
CBP-4	1:1	80	Neutralized phthalate buffer pH 5.0	32	30	30	4.27 ± 0.09
CBR-1	1:0.5	80	Neutralized phthalate buffer pH 5.0	32	30	30	2.10 ± 0.007
CBR-2	1:2	80	Neutralized phthalate buffer pH 5.0	32	30	30	2.24 ± 0.03

**Table 2:** Optimization of CBZ-HP-β-CD complexes using HLE process

Batch	Drug:β-CD molar ratio	Screw speed (rpm)	Solvent composition	Temperature of barrel (°C)			Solubility (mg/mL)
				B2	B3	B4	
Optimization of screw speed							
CHS-1	1:1	60	Water	32	30	30	4.26 ± 0.05
CHS-2	1:1	80	Water	32	30	30	3.4 ± 0.08
CHS-3	1:1	100	Water	32	30	30	4.12 ± 0.02
Optimization of solvent composition							
CHS-1	1:1	60	Water	32	30	30	4.26 ± 0.05
CHP-1	1:1	60	Hydrochloric acid buffer pH 1.2	32	30	30	3.74 ± 0.007
CHP-2	1:1	60	Acetate buffer pH 4.6	32	30	30	6.39 ± 0.09
CHP-3	1:1	60	Phosphate buffer pH 5.8	32	30	30	4.01 ± 0.007
CHP-4	1:1	60	Phosphate buffer pH 6.8	32	30	30	4.10 ± 0.007
CHP-5	1:1	60	Phosphate buffer pH 7.4	32	30	30	3.98 ± 0.03
CHP-6	1:1	60	Alkaline borate buffer pH 9.2	32	30	30	4.49 ± 0.03
Optimization of molar ratio							
CHP-2	1:1	60	Acetate buffer pH 4.6	32	30	30	6.39 ± 0.09
CHR-1	1:0.5	80	Acetate buffer pH 4.6	32	30	30	3.27 ± 0.11
CHR-2	1:2	80	Acetate buffer pH 4.6	32	30	30	4.96 ± 0.09

#### Physical mixture

Drug-cyclodextrin physical mixtures were prepared using β-CD (PMB) or HP-β-CD (PMH). Briefly, 2 g of drug and cyclodextrin (1:1 molar ratio) were blended for 10 min and transferred to an air tight container and stored.

#### Solvent evaporation

Carbamazepine and β-CD or HP-β-CD (1:1 molar ratio; 2 g) was added to 20 mL of ethanol. The resulting mixture in a round bottom flask was evaporated at 60 °C using a rotary flash evaporator (R-215, Buchi, Switzerland) at 40 rpm until all the solvent was evaporated. The residue was scrapped, sieved, and stored.

#### Kneading method

Briefly, CBZ and β-CD or HP-β-CD mixtures (1:1 molar ratio; 2 g) were taken in a mortar and pestle and to it enough neutralized phthalate buffer pH 5.0 (for β-CD; KMB) or acetate buffer pH 4.6 (for HP-β-CD; KMH) was added to form a slurry. The slurry was triturated for 15 min, transferred to a petri plate, and dried at 50 °C for 24 h. The resulting dry residue was sieved through no. 40 mesh and stored in an airtight container.

### Characterization of drug-cyclodextrin complexes

All the prepared complexes were characterized using differential scanning calorimetry (DSC), Fourier transform infrared spectroscopy (FTIR), and X-ray diffraction (XRD) studies. The samples which were analyzed consisted of plain carbamazepine (CBZ), beta-cyclodextrin (β-CD), hydroxypropyl beta-cyclodextrin (HP-β-CD), physical mixture of CBZ and β-CD (PMB; 1:1 ratio), physical mixture of CBZ and HP-β-CD (PMH; 1:1 ratio), optimized complexes prepared by HLE technique (CBP-4 and CHP-2), and complexes prepared by kneading method (KMB and KMH).

### In vitro drug release studies

Release profile of CBZ and optimized complexes (CBP-4 and CHP-2) was studied using USP Type-2 apparatus (Electrolab TDT-08L DT, Mumbai, India). Accordingly, complexes containing 100 mg equivalent of carbamazepine was weighed and added into the dissolution jar. Dissolution medium consisted of phosphate buffer pH 6.8 (900 mL). The dissolution apparatus was set at 37 ± 0.5 °C. The paddle was operated at a constant speed of 75 rpm. At time intervals of 5, 10, 15, 20, 30, 40, 50, and 60 min, samples (5 mL) were withdrawn from the dissolution jar and filtered using 0.45-μ filter. The volume withdrawn from the dissolution jar was replaced with an equal volume of fresh media to preserve the sink conditions. The amount of CBZ was analyzed using HPLC.

### In vivo pharmacokinetic studies

Wistar rats weighing 200–250 g were used to carry out the pharmacokinetic studies. The animals were divided into 3 groups, each containing 6 animals. Each group was administered orally with different formulations as mentioned below:

1. Group 1—plain carbamazepine (18 mg/kg)
2. Group 2—CBZ-HP-β-CD complex prepared by kneading method (KMH)
3. Group 3—optimized CBZ-HP-β-CD complex prepared by HLE method (CHP-2)

Drug or formulations (equivalent to 18 mg/kg of CBZ) were dispersed in 0.5% sodium carboxymethylcellulose and administered at volumes less than 1.0 mL. At time intervals between 0 and 72 h, about 500 μL of blood was withdrawn from retro-orbital plexus of each rat and centrifuged at 8000 rpm for 5 min to separate the plasma. The amount of drug present in the samples was determined using HPLC. Winnonlin software was used to calculate the pharmacokinetic parameters from the data obtained. The experimental pharmacokinetic data was statistically analyzed by one-way ANOVA followed by Tukey’s post hoc tests using Graph Pad Prism Software. *p* value less than 0.05 was considered statistically significant.

## Results and discussion

### Saturation solubility of CBZ

Solubility of CBZ was carried out in water or standard buffer solutions of varying pH. The solubility of CBZ was observed to be 0.140 ± 0.0007 mg/mL in water, 0.148 ± 0.0007 mg/mL in pH 1.2 hydrochloric acid buffer, 0.206 ± 0.0007 mg/mL in pH 4.6 acetate buffer, 0.136 ± 0.0007 mg/mL in pH 5.8 phosphate buffer, 0.140 ± 0.0007 mg/mL in pH 6.8 phosphate buffer, 0.111 ± 0.0007 mg/mL in pH 7.4 phosphate buffer, and 0.137 ± 0.0016 mg/mL in pH 9.2 alkaline borate buffer. Carbamazepine is categorized as a class II drug as per the Biopharmaceutical Classification System (BCS), displaying low solubility and high permeability. The solubility of CBZ in each of the media did not vary significantly indicating that carbamazepine exhibits pH-independent solubility. This shows that CBZ is essentially a neutral molecule without acid or base characteristics in the entire pH range of 2 to 9 [40].

### Phase solubility studies

**Fig. 2:**
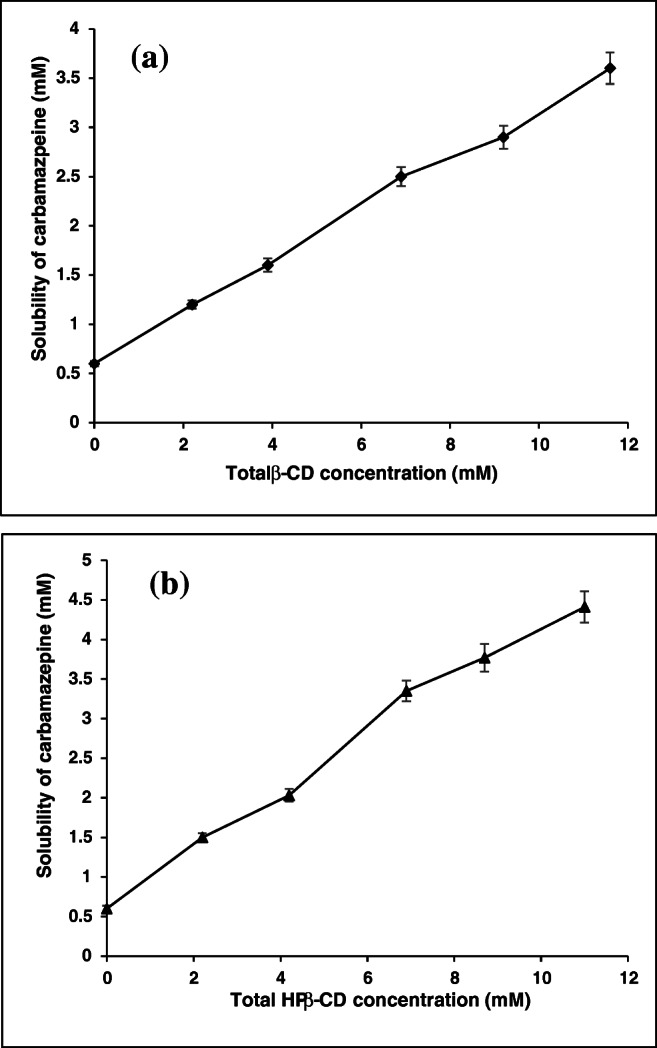
Phase solubility diagram of **a** CBZ:β-CD and **b** CBZ:HP-β-CD at different molar concentrations in aqueous media

The phase solubility profiles of CBZ-β-CD and CBZ-HP-β-CD are shown in Fig. 2a and b. The solubility of CBZ was observed to increase with increase in concentrations of CDs and hence A_L_-type solubility profile was observed in both the cases [39, 41]. From the phase solubility diagrams for β-CD and HP-β-CD, the stability constants (*K*_s_) were found to be 574 M^−1^ and 899 M^−1^, and the host-guest correlation coefficient (*R*^2^) values were 0.9902 and 0.9954. The slopes were found to be 0.256 for CBZ-β-CD and 0.3506 for CBZ-HP-β-CD which is less than 1 indicating the formation of a complex having 1:1 molar ratio. In addition, the complexation efficiency (CE) of CBZ-β-CD and CBZ-HP-β-CD was found to be 0.345 and 0.546. All these results clearly indicate that complexes prepared using HP-β-CD are more stable. These results are corroborated by similar data obtained in previous reports [42].

### Preparation and optimization of drug-cyclodextrin complexes

#### Hot liquid extrusion using twin-screw melt extruder

Drug-cyclodextrin complexes were prepared by hot liquid extrusion using a twin-screw hot melt extruder and the process was optimized with respect to screw speed, solvent composition, temperature of barrel, and molar ratio. Optimization was done by varying each parameter at a time while keeping other parameters constant and comparing the solubility of the complexes with that of plain CBZ (Tables 1 and 2).

Initially, the effect of screw speed of the melt extruder processor was evaluated. Though CBS-9 complex, prepared at 250 rpm, showed highest solubility (2.11 ± 0.01 mg/mL) among all the β-CD complexes prepared, this speed was not selected due to potential detrimental effect on the formation of complexes. CBS-5 complex prepared at a screw speed of 80 rpm showed a solubility of 1.87 ± 0.03 mg/mL. The results from solubility studies clearly indicate that CBS-5 exhibited nearly 12 times higher solubility as compared to plain CBZ. This could be because the drug had sufficient time to get kneaded at 80 rpm and enter inside the cyclodextrin cavity to form the inclusion complex. In the case of HP-β-CD complexes, operating the screw at 60 rpm (CHS-1) was found to form complexes with greater solubility (4.26 ± 0.05 mg/mL) compared to other screw speeds.

Among the different media used for the preparation of CBZ-β-CD complexes, it was observed that complexes prepared using neutralized phthalate buffer pH 5.0 showed higher solubility (CBP-4; 4.27 ± 0.09 mg/mL) compared to other solvents. Preparation of CBZ-HP-β-CD complexes using acetate buffer pH 4.6 (CHP-2) showed better solubility (6.39 ± 0.09 mg/mL) as compared to other buffer systems. This could be ascribed to lesser ionization of drug at these pH values and hence greater hydrophobicity of drug leading to better complexation of drug into cyclodextrin cavity to form inclusion complex [40].

Previous reports have shown that the effect of temperature on drug-CD complexation may be negligible when the interaction is due to release of water molecules between the host and guest entities [43]. Accordingly, drug-cyclodextrin complexes prepared at room temperature were selected (B2:32 °C, B3:30 °C, B4:30 °C) because selecting higher temperatures may prove detrimental to the formulation. Moreover, there was no much difference in the solubility of the drug from the complexes prepared at elevated temperatures.

Preparing complexes at varying drug:cyclodextrin ratios indicated that complexes prepared at 1:1 molar ratio (CBP-4 and CHP-2) showed higher solubility as compared to complexes prepared at other molar ratios.

The optimized complexes CBP-4 and CHP-2 showed a solubility of 4.27 ± 0.09 mg/mL and 6.39 ± 0.09 mg/mL respectively as compared to plain CBZ, which showed a solubility of 0.140 ± 0.007 mg/mL. It is interesting to note that the solubility of CHP-2 complex was nearly forty-five times higher than the solubility of plain CBZ and nearly one-and-a-half times the solubility of CBP-4 complex. This clearly indicates the higher solubility of CBZ-HP-β-CD complex over CBZ-β-CD complex.

#### Physical mixture

Drug-cyclodextrin physical mixtures prepared using β-CD (PMB) or HP-β-CD (PMH) were subjected to solubility studies. The solubility of PMB complex was found to be 1.22 ± 0.11 mg/mL and that of PMH complex was found to be 3.9 ± 0.06 mg/mL.

#### Solvent evaporation

Drug-CD complexes prepared by solvent evaporation method showed a solubility of 1.96 ± 0.02 mg/mL (CBZ-β-CD) and 4.23 ± 0.04 mg/mL (CBZ-HP-β-CD). Clearly, these results indicated the advantage of complexes prepared by HLE process (CBP-4 and CHP-2) in enhancing the solubility of CBZ.

#### Kneading method

Among the conventional methods, complexes prepared using kneading method showed lower solubility (KMB 2.52 ± 0.007 mg/mL, KMH 4.92 ± 0.05 mg/mL) of the drug as that observed with extrusion process (CBP-4 and CHP-2). This could be because during kneading, the drug would have enough time to get loaded into the CD cavity than compared to other conventional methods like solvent evaporation and physical mixture. Presence of water in the preparation of complexes, as observed in kneading method, greatly increases the chances of successful complexation [14]. Higher hydrophilicity and wetting property due to superior interaction between drug and CD during the process of kneading may be responsible for higher solubility of complexes as compared to those prepared by solvent evaporation method [16]. Hence, it can be construed that the complexes prepared by HLE process as a method of preparation of drug-CD complexes were much better than kneading method and can be used as an effectual method to prepare complexes. Based on the above results, CBP-4 and CHP-2 were selected to be used for further studies.

### Characterization of drug-cyclodextrin complexes

#### Fourier transform infrared spectroscopy studies

**Fig. 3:**
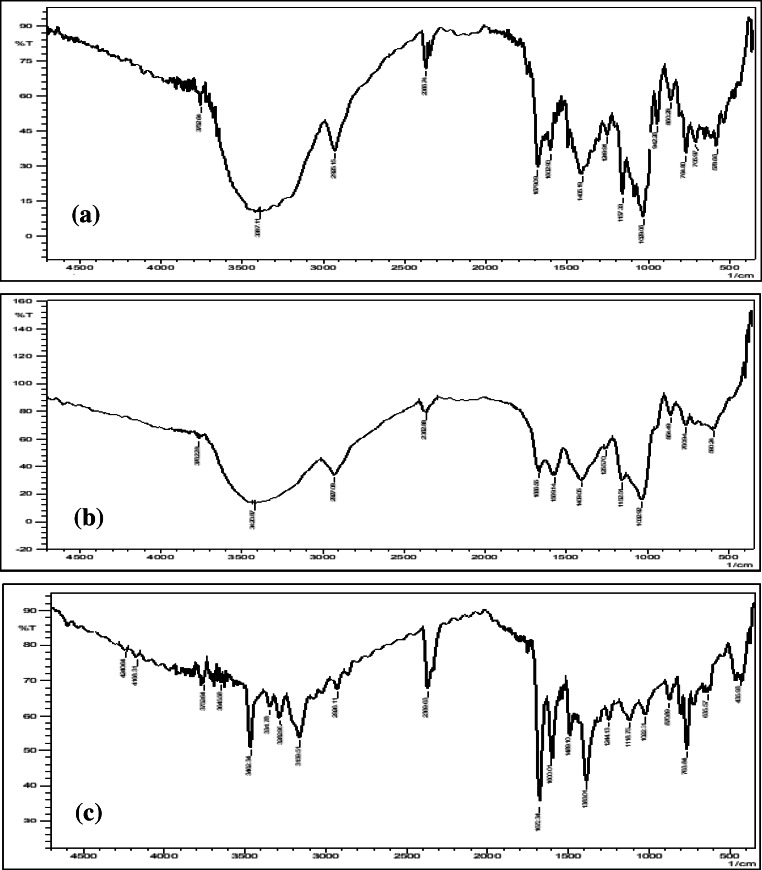
IR spectra. **a** Plain carbamazepine (CBZ), **b** optimized CBZ-β-CD complex prepared by HLE process (CBP-4), and **c** Optimized CBZ-HP-β-CD complex prepared by HLE process (CHP-2)

The IR spectrum of CBZ (Fig. 3a) exhibited major peaks at 3462.34 cm^−1^ (–NH stretching vibrations), 1672.34 cm^−1^ and 1600.01 cm^−1^ (strong –C=O and –C=C– vibrations), and 1383.01 cm^−1^ (–NH deformation) [25]. β-CD spectrum (Fig. S1.A) exhibited a broad and large peak at 3391.94 cm^−1^ (strong –OH stretching vibration), 2922.25 cm^−1^ (medium –CH_2_ stretching vibration), 1156.36 cm^−1^ (–C–C– vibrations), and 1029.08 cm^−1^ (–OH bending vibration) [44]. The IR spectrum of HP-β-CD shown in (Fig. S1.B) exhibited broad and low intense peaks at 3413.15 cm^−1^ (–OH stretching vibration), 1643.41 cm^−1^ (H–O–H bending vibration), and 1158.29 cm^−1^ (asymmetrical C–O–C stretching vibration) [45].

The IR spectrum of the physical mixture of CBZ and β-CD (PMB; 1:1 ratio), (Fig. S1.C) imitated the characteristic bands of CBZ. The frequency and the intensities of the major drug peaks remained unaltered indicating plain superimposition of the bands of host and guest molecules [22]. CBZ and HP-β-CD physical mixture (PMH; 1:1 ratio) exhibited an IR spectrum (Fig. S1.D) that retained the peaks of the drug. In other words, the IR spectrum of the physical mixture was a simple overlap of HP-β-CD and CBZ bands [46].

The analysis of the IR spectrum of CBZ-β-CD complex (Fig. S1.E) prepared by kneading method (KMB) showed a shift in the bands of CBZ towards higher frequency as is indicated by the low intense peak at 1688.73 cm^−1^ of the carbonyl function and a broad, low intense peak at 3445.94 cm^−1^ of –NH valence. Analogous results were observed in the IR spectrum of complex prepared using HLE technique (CBP-4; Fig. 3b) where the characteristic –NH stretch shifted to 3387.11 cm^−1^ and the strong carbonyl stretching vibration shifted to a less intense peak at 1679.08 cm^−1^. The decrease in intensity of bands could be due to the dissociation of intermolecular H bonds between CBZ molecules indicative of interaction with cyclodextrin resulting in its enclosure within the hydrophobic cavity [47, 48].

The IR spectrum of CBZ-HP-β-CD complex prepared by kneading method (KMH) and HLE (CHP-2) (Fig. S1.F and Fig. 3c) gave rise to some interesting observations. The spectrum of KMH showed a shift in the peak corresponding to the –NH valence to a lower frequency (3414.12 cm^−1^) whereas the carbonyl function shifted to a less intense peak at 1688.73 cm^−1^. Likewise, the IR spectrum of CHP-2 also showed a similar trend with peaks at 3387.11 cm^−1^ and 1679.08 cm^−1^. These results can be attributed to formation of hydrogen bonding between the carbonyl function of CBZ and the hydroxyl groups present in the cyclodextrin cavity as well as presence of van der walls forces therefore indicating the formation of CBZ-HP-β-CD inclusion complex [48].

#### Differential scanning calorimetry

**Fig. 4:**
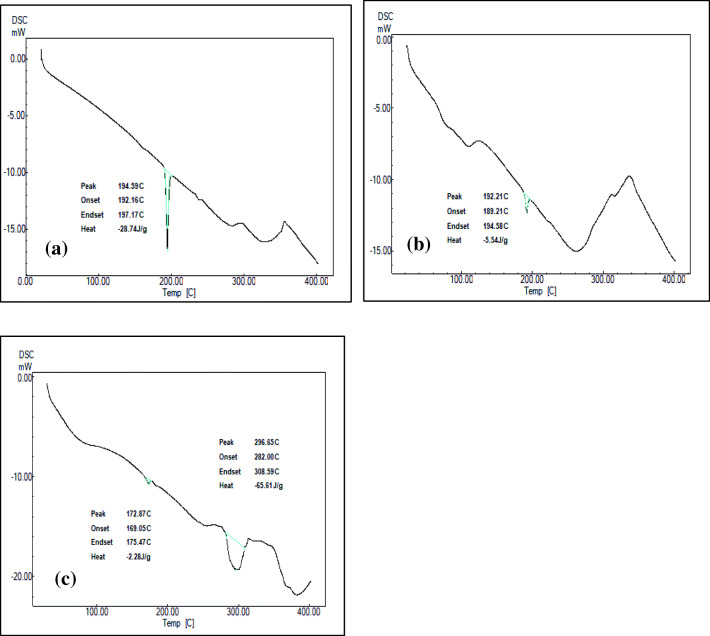
DSC thermograms. **a** Plain carbamazepine (CBZ), **b** optimized CBZ-β-CD complex prepared by HLE process (CBP-4), and **c** optimized CBZ-HP-β-CD complex prepared by HLE process (CHP-2)

Differential scanning calorimetry (DSC) as a standalone analytical technique can indicate the phase transition or thermal stability of drug-cyclodextrin complexes. However, in combination with crystallographic analysis like XRD, a more detailed and definitive evidence regarding the physical properties of the solid-state complexes can be gathered. DSC thermogram of plain CBZ, shown in Fig. 4a, exhibited a strong endothermic peak in accordance with its melting point, at 194.59 °C (∆H = − 28.74 J/g) and indicating its crystalline anhydrous nature. The DSC thermogram of β-CD and HP-β-CD (Fig. S2.A and B) exhibited broad endothermic peak in the range of 90–130 °C and at above 300 °C corresponding to dehydration followed by decomposition. DSC curve of the physical mixture of CBZ and β-CD (PMB; Fig. S2.C) showed the characteristic drug melting peak, however with a low intensity, and was a sum of the thermograms obtained with CBZ and β-CD (∆H = − 8.56 J/g). Similar results were observed with DSC curve of the physical mixture of CBZ and HP-β-CD (PMH; ∆H = − 2.22 J/g) (Fig. S2.D). DSC thermogram of β-CD complex prepared by kneading method (KMB; Fig. S2.E) resulted in the presence of the drug melting peak albeit low in intensity (∆H = − 5.54 J/g). In the same way, DSC thermogram of optimized complex CBP-4 (Fig. 4b) prepared using HLE showed retention of the peak of the drug with decreased intensity. Usually complexation of drug with cyclodextrin results in either shifting of the drug peak to lower temperature or its complete disappearance signifying a loss in the crystallinity of the drug molecule [48, 49]. On the other hand, retention of drug peak, albeit low in intensity, may indicate an incomplete formation of inclusion complex. To this end, the relative drug crystallinity (RDC) of the complexes can be used to assess drug-CD interactions. The ratio of the melting enthalpy (∆H) of the sample (physical mixture or complex) and that of the pure drug indicates the extent of crystallinity of the interacted mixture. This explains the presence of residual drug peak in the case of complexes KMB and CBP-4 with %RDC values of 27.20 and 19.27 respectively and hence is indicative of the amorphizing nature of cyclodextrins. Also, it is interesting to note that complexes prepared using HLE technique (CBP-4) were relatively more amorphous compared to complexes prepared by kneading method (KMB).

However, in the case of HP-β-CD complexes, the crystalline drug peak in the DSC thermograms of CHP-2 and KMH complex completely disappeared (Fig. 4c and Fig. S2.F) indicating a total drug amorphization due to higher affinity of solid-solid interactions of HP-β-CD with CBZ. All these results suggest that HLE technique also produces drug-CD complexes which are comparable to the complexes prepared by kneading method. However, a conclusive confirmation regarding the formation of solid-state complexes can be obtained using an XRD analysis.

#### X-ray diffraction analysis

**Fig. 5:**
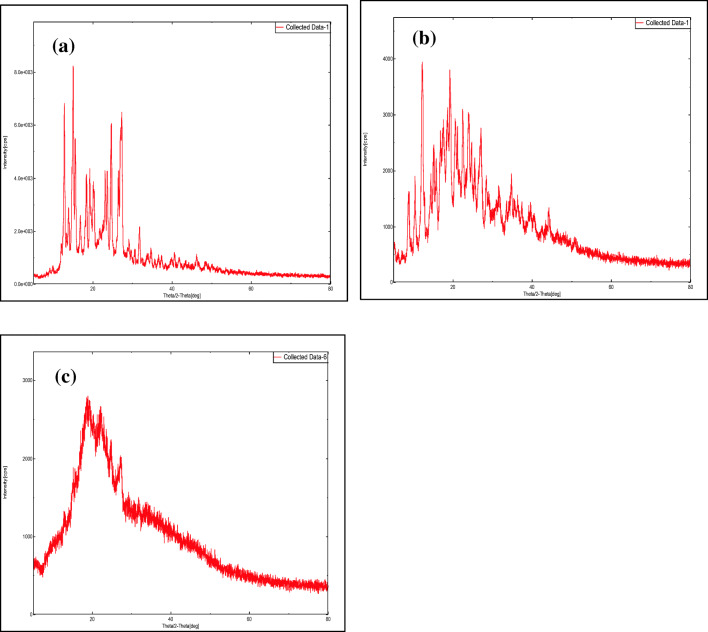
XRD patterns. **a** Plain carbamazepine (CBZ), **b** optimized CBZ-β-CD complex prepared by HLE process (CBP-4), and **c** optimized CBZ-HP-β-CD complex prepared by HLE process (CHP-2)

The X-ray diffraction patterns of plain CBZ, β-CD, HP-β-CD, and physical mixtures PMB and PMH; optimized complexes CBP-4 and CHP-2; and complexes KMB and KMH are shown in Fig. 5a to c and Figs. S3.A to S3.F. The XRD pattern of pure CBZ showed sharp peaks at 2θ angles 14.99°, 15.54°, 19.17°, 24.60°, and 26.94° indicating the highly crystalline nature of carbamazepine [50]. The crystalline nature of β-CD was apparent from its XRD pattern which showed sharp peaks at 12.21°, 12.48°, and 22.44° while the XRD pattern of HP-β-CD exhibited broad and diffused peaks at 11.51° and 18.44° indicating its amorphous nature [22, 51]. The XRD pattern of PMB and PMH combined the peaks of drug and β-CD or HP-β-CD, however with reduced intensities. Complex KMB and CBP-4 showed broad but fewer peaks corresponding to the drug with low intensities. This decrease in number and intensity of peaks could be due to the presence of the drug in a relatively amorphous matrix. Similar results were observed with complex KMH and CHP-2 which showed broad and diffused peaks indicating amorphization of the crystalline drug within the inclusion complex [22, 48].

### In vitro drug release studies

**Fig. 6:**
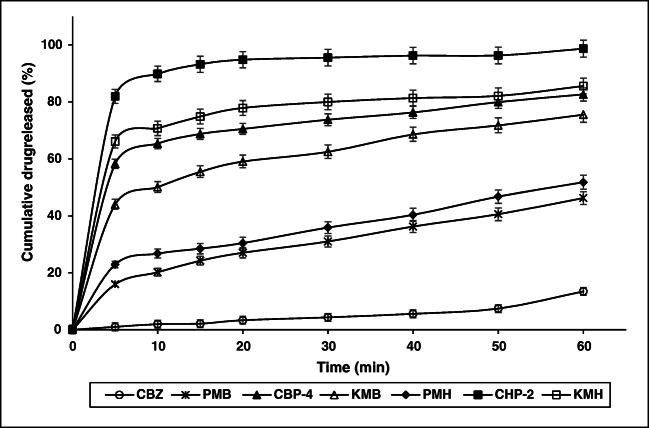
In vitro drug release studies of pure CBZ, and its physical mixtures and complexes. CBZ: carbamazepine; PMB: physical mixture of CBZ and β-CD (1:1 ratio); PMH: physical mixture of CBZ and HP-β-CD (1:1 ratio); KMB: CBZ-β-CD complex prepared by kneading method; KMH: CBZ-HP-β-CD complex prepared by kneading method; CBP-4: optimized CBZ-β-CD complex prepared by HLE; CHP-2: optimized CBZ-HP-β-CD complex prepared by HLE

The in vitro drug release patterns of pure CBZ, its physical mixtures with β-CD and HP-β-CD, complexes prepared using conventional method (kneading), and the optimized complexes prepared using HLE are presented in Fig. 6. Plain carbamazepine showed a cumulative drug release of only 13.47 ± 0.54% at the end of 60 min. In contrast, physical mixtures PMB and PMH showed manifold increase in dissolution of CBZ which could be attributed to the hydrophilicity of the cyclodextrin molecule that offers enhanced drug solubility. Further, cyclodextrin complexes prepared using kneading method, i.e., KMB and KMH, also exhibited a significant increase in CBZ dissolution (75.52 ± 2.68% and 85.59 ± 2.80%). Pertaining to the complexes prepared by HLE process (CBP-4 and CHP-2), more than 50% of CBZ was released in just 5 min compared to a release of 1% from plain CBZ. Among the CD complexes, CHP-2 showed higher release of CBZ (~ 80%) in just 5 min as compared to CBP-4 (~ 58%). This can be attributed to the higher solubility of CHP-2 as compared to CBP-4 which typically enhanced the dissolution of CHP-2. At the end of 1 h, cumulative drug release observed from CHP-2 was 98.69 ± 2.96% and that of CBP-4 was found to be 82.64 ± 2.96%. Hence, CHP-2 was considered for further pre-clinical pharmacokinetic studies. In general, complexes prepared using HP-β-CD (physical mixture, kneading, or HLE) showed better drug release which could be ascribed to its enhanced solubility and the resultant better dissolution. It is interesting to note that the complexes prepared using HLE (CBP-4 or CHP-2) showed significantly higher drug release as compared to a conventional method like kneading (KMB or KMH). In vitro dissolution studies clearly indicate the formation of an inclusion complex of CBZ with CDs using HLE which can be accounted for the resultant superior dissolution rates. Also, the fine intermeshing between the rotating screws of the extruder provides a mechanical force that ensures homogenous distribution of CBZ in the CD matrix resulting in the transformation of crystalline drug to an amorphous state which in turn enhances the solubility of CBZ. Previously, Yano and Kleinebudde [52] reported the application of a wet extrusion technique to enhance the dissolution behavior of indomethacin using CD. Drug-CD interaction coupled with the increased wettability due to association of drug and CD by the agitating force offered by the rotating screws of the extruder significantly enhance the dissolution of the drug. Hence, HLE can be considered an effective method for the preparation of CBZ-CD inclusion complexes.

### In vivo pharmacokinetic studies

**Fig. 7:**
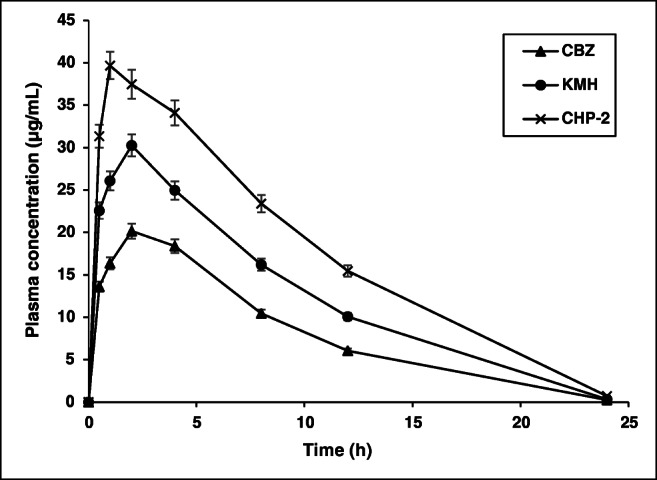
Plasma concentration versus time profile obtained in pharmacokinetic studies (mean ± SD; *n* = 6). CBZ: carbamazepine; KMH: CBZ-HP-β-CD complex prepared using kneading method; CHP-2: optimized CBZ-HP-β-CD complex prepared using HLE

In vivo pharmacokinetic studies were carried out in Wistar rats for plain drug, complex prepared using kneading method (KMH), and optimized complex prepared using HLE (CHP-2) following oral administration. The mean pharmacokinetic parameters are shown in Table 3 and the plasma concentration versus time profile is shown in Fig. 7. Plain drug exhibited a maximum concentration (*C*_max_) of 20.1 ± 0.46 μg/mL. In contrast, KMH complex showed ~ 1.5-fold increase (30.3 ± 0.70 μg/mL) (*p* < 0.05) whereas CHP-2 demonstrated ~ 2-fold increase (39.7 ± 0.91 μg/mL) in *C*_max_ (*p* < 0.05). The time required to achieve maximum concentration (*T*_max_) was observed to be the same for both plain drug and KMH complex (2 ± 0.00 h) while in the case of CHP-2, *T*_max_ was achieved within 1 ± 0.00 h itself. Also, CHP-2 complex showed a longer retention time (MRT = 6.93 ± 0.22 h) (*p* < 0.05) as compared to plain CBZ (6.5 ± 0.20 h) and KMH (6.6 ± 0.20 h). A significant increase in AUC_0–24_ was observed with CHP-2 complex (425.25 ± 12.76 h·μg/mL) (*p* < 0.05) when compared to plain CBZ and KMH (*p* < 0.05) complex. This could be attributed to the close intermeshing provided by the co-rotating screws of the extruder coupled with the higher solubility of HP-β-CD complexes which resulted in higher absorption of CBZ [53]. The elimination rate constant (Ke) was low for plain CBZ (0.264 ± 0.01 h^−1^) as well as complexes (KMH 0.29 ± 0.01 h^−1^; CHP-2 0.26 ± 0.01 h^−1^). Also there was a significant difference in the *t*_1/2_ values of KMH and CHP-2 when compared to plain CBZ (*p* < 0.05). It is quite evident from the above results that pharmacokinetic parameters required to assess absorption (such as AUC, *T*_max_ and *C*_max_) were significantly higher than those for plain drug as well as complex prepared using kneading method (KMH). These results corroborate the results obtained from in vitro release studies. In a previous study reported by Choudhury and Nelson [22], in vivo pharmacokinetic studies of plain CBZ revealed similar results when compared with drug-cyclodextrin complexes. A 2-fold increase was observed in *C*_max_ and AUC_0–12_, indicating the bioavailability of drug-CD complexes was more compared to plain CBZ due to increased solubility and hence faster dissolution, which further reaffirms the results of in vivo pharmacokinetics in the present study. Hence, this reiterates the advantage of HLE process over and above the conventional kneading method in the preparation of CBZ-CD complexes. Moreover, the HLE process used in the present study also avoids the classical melting approach for drug/CD thereby minimizing exposure to heat.

**Table 3:** Pharmacokinetic parameters of carbamazepine (CBZ), complex prepared by kneading method (KMH), and complex prepared by HLE (CHP-2) when administered orally to Wistar rats

Parameters	CBZ	KMH	CHP-2
*C*_max_ (μg/mL)	20.1 ± 0.46	30.3 ± 0.70*	39.7 ± 0.91*#
*T*_max_ (h)	2 ± 0.00	2 ± 0.00	1 ± 0.00*#
AUC_0–24_ (h·μg/mL)	196 ± 6.08	298.30 ± 9.25*	425.25 ± 12.76*#
AUC_0–∞_ (h·μg/mL)	196.9 ± 6.10	299.50 ± 9.28*	427.95 ± 12.84*#
*t*_1/2_ (h)	2.62 ± 0.08	2.43 ± 0.08*	2.68 ± 0.07#
Ke (1/h)	0.264 ± 0.01	0.29 ± 0.01*	0.26 ± 0.01#
MRT (h)	6.5 ± 0.20	6.6 ± 0.20	6.93 ± 0.21*#

All values are expressed as mean ± SD; *n* = 6

*AUC* area under the curve, *t*_*1/2*_ elimination half-life, *Ke* elimination rate constant, *MRT* mean residential time

*p value less than 0.05 (statistically significant) in comparison with CBZ

^#^*p* value less than 0.05 (statistically significant) in comparison with KMH

## Conclusion

Drug-CD complexes were prepared using conventional methods as well as by HLE process using a twin-screw melt extruder. Among the complexes prepared by HLE process, CBZ-HP-β-CD complex showed manifold increase in solubility compared to plain drug and CBZ-β-CD complex, and over complexes prepared by a conventional method like kneading method. Further, in vitro drug release studies indicated higher release of CBZ from complexes prepared by HLE compared to plain drug and kneading method, clearly demonstrating the advantage of HLE process in the preparation of CBZ-CD complexes. In addition, CHP-2 complex showed the highest drug release compared to CBP-4 owing to superior solubility. Pharmacokinetic parameters also revealed a significant improvement in the dissolution behavior of CBZ and hence a higher bioavailability compared to plain CBZ and complex prepared by kneading (KMH). Overall, a 2-fold increase in solubility and bioavailability of CBZ was observed with HLE compared to kneading method, which confirms our hypothesis that HLE is an effectual method in the preparation of drug loaded CD complexes.

## Electronic supplementary material

ESM 1 (DOCX 250 kb)
