# Supplementary material for: Hot liquid extrusion assisted drug-cyclodextrin complexation: a novel continuous manufacturing method for solubility and bioavailability enhancement of drugs
Source: Drug Deliv Transl Res. 2020 Sep 21;11(3):1273–87. doi: 10.1007/s13346-020-00854-w (PMC8096738; doi:10.1007/s13346-020-00854-w)
Supplement: Supplementary file 1 — (DOCX 250 kb) [file 13346_2020_854_MOESM1_ESM.docx]

**SUPPLEMENTARY INFORMATION**

**
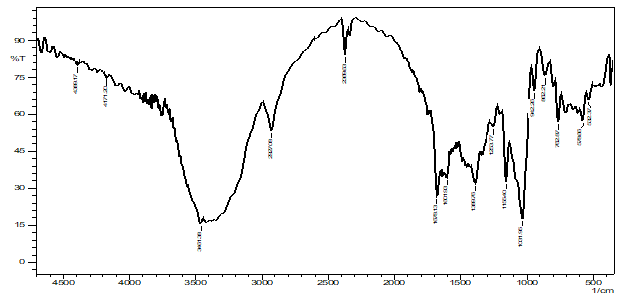

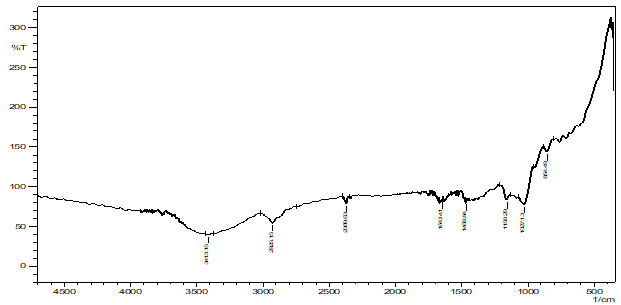

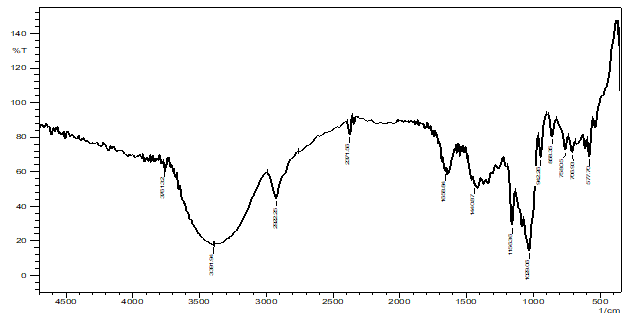
**

**C)**

**B**

**A**

**
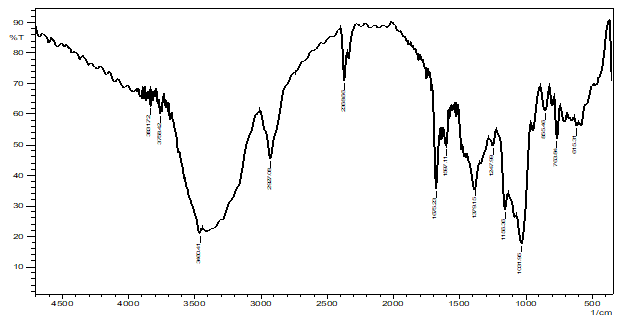

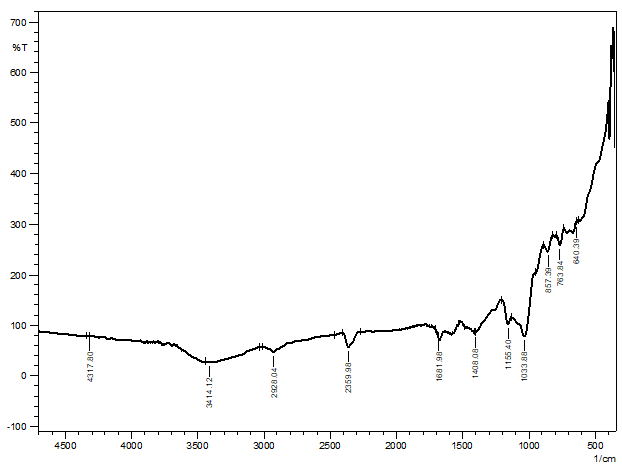

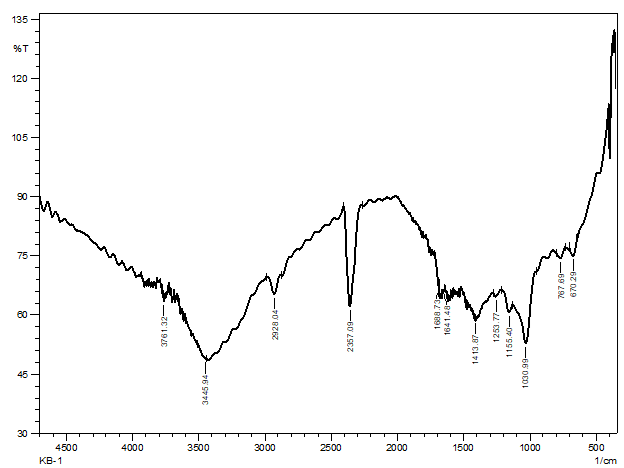
**

**F**

**E**

**D**

**Fig. S1. IR Spectra**

(A) Beta-cyclodextrin (β-CD), (B) hydroxypropyl beta-cyclodextrin (HP-β-CD), (C) physical mixture (PMB), (D) Physical mixture (PMH), (E) complex prepared by kneading method (KMB), F) complex prepared by kneading method (KMH).

**
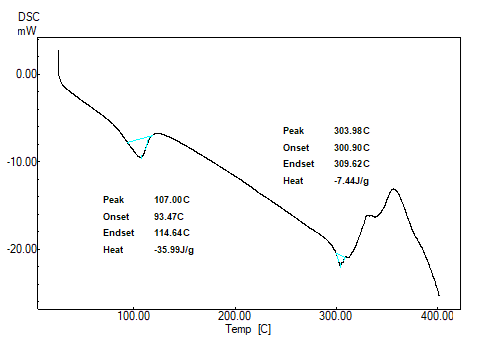
**
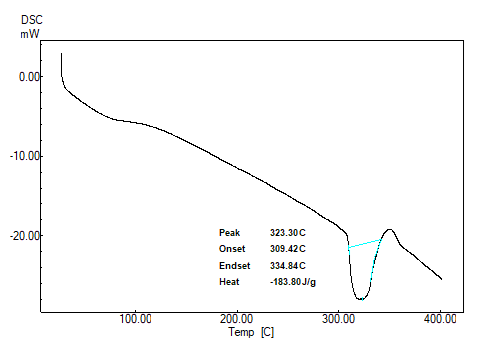


**B**

**A**


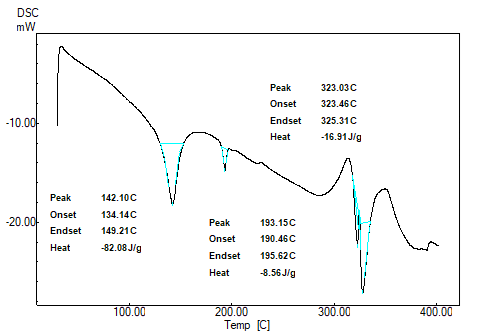

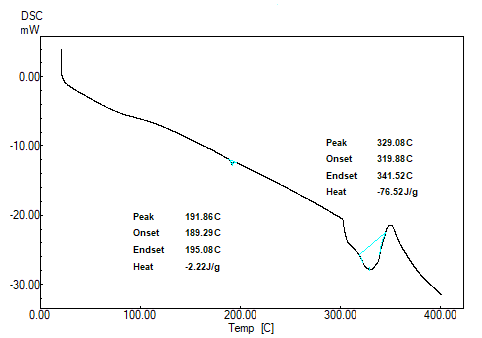


**D**

**C**


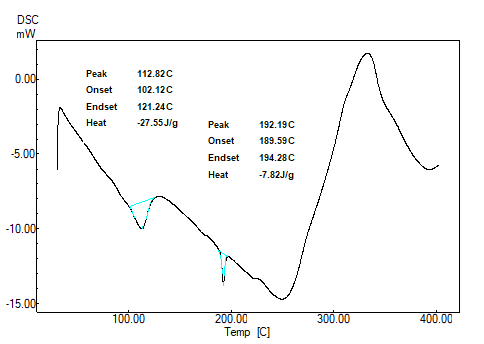

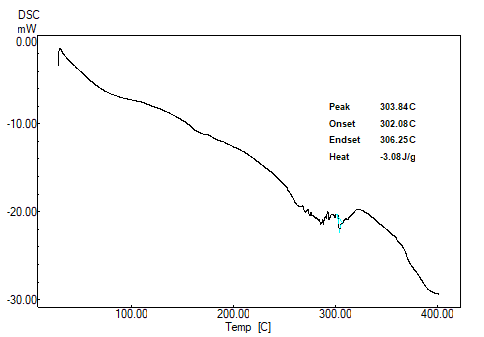


**F**

**E**

**Fig. S2. DSC thermograms**

(A) Beta-cyclodextrin (β-CD), (B) hydroxypropyl beta-cyclodextrin (HP-β-CD), (C) physical mixture (PMB), (D) Physical mixture (PMH), (E) complex prepared by kneading method (KMB), F) complex prepared by kneading method (KMH).

**B**

**A**

**D**

**C**

**F**

**E**

**Fig. S3. XRD patterns**

(A) Beta-cyclodextrin (β-CD), (B) hydroxypropyl beta-cyclodextrin (HP-β-CD), (C) physical mixture (PMB), (D) Physical mixture (PMH), (E) complex prepared by kneading method (KMB), F) complex prepared by kneading method (KMH).
